# Supplementary material for: Structural elements required for the efficient loading and activation of HELB on RPA-coated single-stranded DNA
Source: bioRxiv. 2026 Jul 17:2026.07.16.738708. Preprint. [Version 1] doi: 10.64898/2026.07.16.738708 (PMC13404968; doi:10.64898/2026.07.16.738708)
Supplement: 1 [file NIHPP2026.07.16.738708v1-supplement-1.pdf]

bioRxiv preprint doi: https://doi.org/10.64898/2026.07.16.738708; this version posted July 17, 2026. The copyright holder for this preprint (which was not certified by peer review) is the author/funder, who has granted bioRxiv a license to display the preprint in perpetuity. It is made available under a CC-BY 4.0 International license.

SUPPLEMENTARY FIGURES

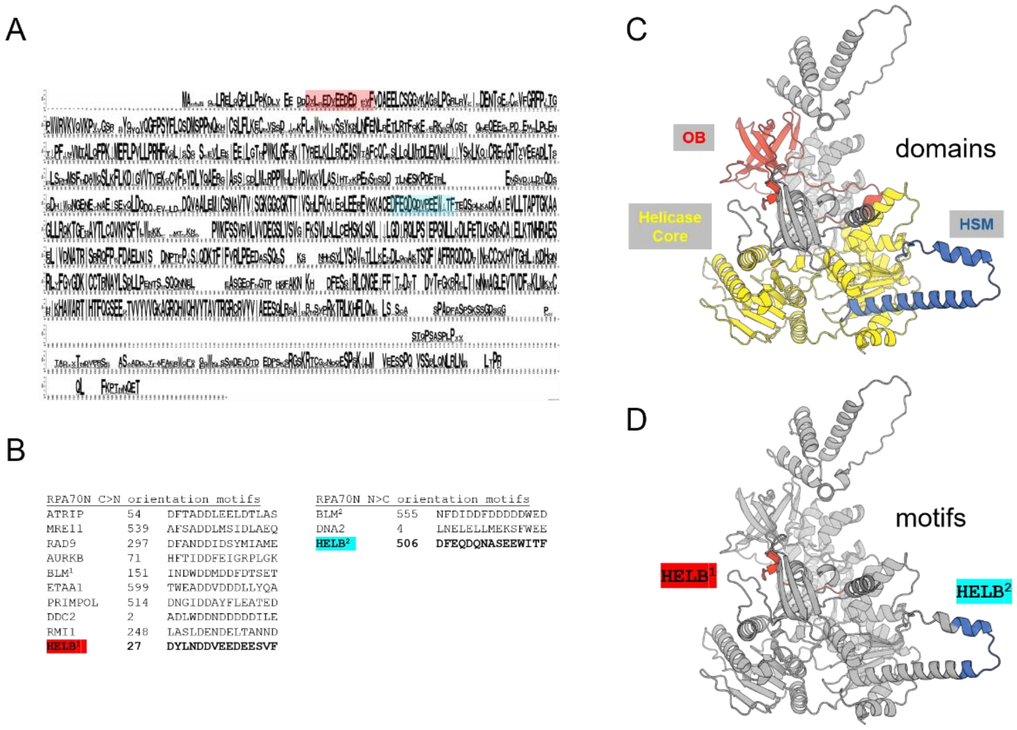

Figure S1: Putative RPA interaction motifs in HELB. (A) Possible RPA interaction motifs (highlighted red and blue) within a multiple sequence alignment of HELB homologues in WebLogo format [41]. To make the Weblogo, fifty representative HELB homologues were retrieved from the UniRef90 database using an E value cutoff of 1e^{-5} and then aligned in COBALT [42, 43]. (B) These conserved motifs contain many basic and hydrophobic residues which is characteristic of proteins that interact with the N-terminal region of RPA70. Note that binding of such peptides to the RPA70N domain has been shown to occur in diverse modes and in two orientations denoted here as N>C and C>N [25]. Therefore, the alignments between peptides are not necessarily intended to indicate conservation of function for individual residues. There is existing experimental support for the role of the HELB^2 motif (blue) motif in RPA interaction [6, 25]. (C) AlphaFold3 model of HELB highlighting an OB-domain (red), the helicase core domains (yellow) and the HELB Specific Motif (HSM) (blue). (D) AlphaFold3 model of HELB highlighting positions of the putative RPA interaction motifs (colour-coding is as in panels A and B). The HELB^1 motif is part of a short insert within the OB domain and the HELB^2 motif resides ta the distal tip of the HSM domain.

bioRxiv preprint doi: https://doi.org/10.64898/2026.07.16.738708; this version posted July 17, 2026. The copyright holder for this preprint (which was not certified by peer review) is the author/funder, who has granted bioRxiv a license to display the preprint in perpetuity. It is made available under a CC-BY 4.0 International license.

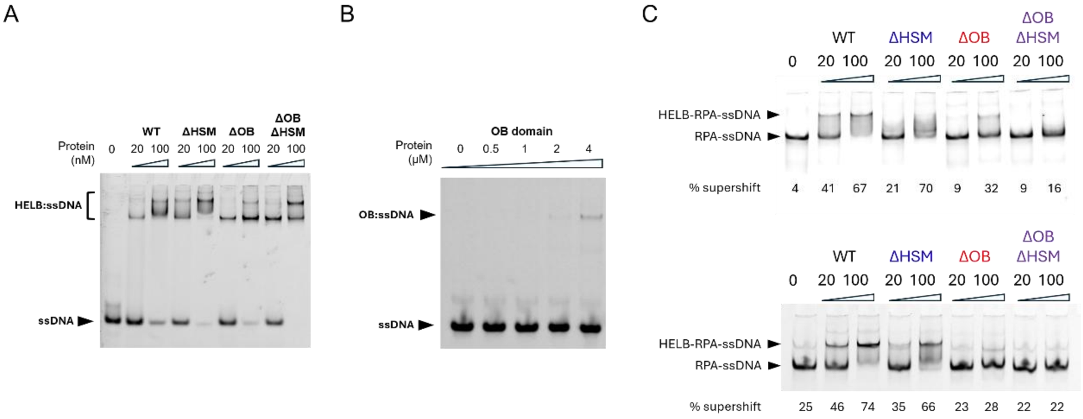

Figure S2: Further examples of ssDNA EMSA and RPA:ssDNA supershift assays for HELB variants. (A) Additional example of HELB EMSA experiment (equivalent to main paper Figure 2A). The gel shows binding of a 25mer ssDNA oligonucleotide (5 nM) by wild-type and variant HELB proteins at the concentrations indicated. (B) Additional example of HELB-OB EMSA experiment (equivalent to main paper Figure 2B). The gel shows binding of a 25mer ssDNA oligonucleotide (5 nM) by HELB-OB domain at the concentrations indicated. (C) Additional examples of HELB RPA:ssDNA complex supershifts (equivalent to main paper Figure 5C). The percentage of supershifted material is shown to provide a quantitative measure of the efficacy of HELB binding to RPA-ssDNA.

bioRxiv preprint doi: https://doi.org/10.64898/2026.07.16.738708; this version posted July 17, 2026. The copyright holder for this preprint (which was not certified by peer review) is the author/funder, who has granted bioRxiv a license to display the preprint in perpetuity. It is made available under a CC-BY 4.0 International license.

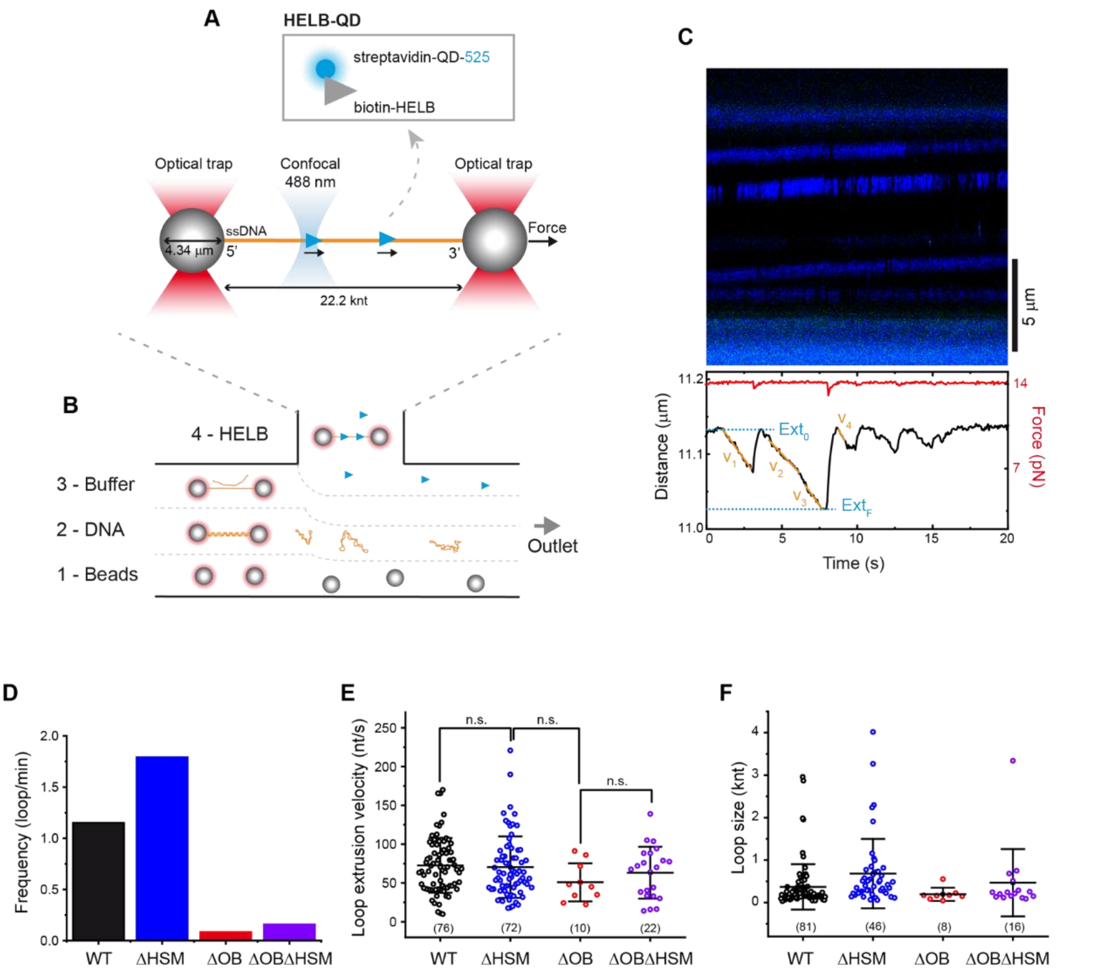

Figure S3: Schematic representation of the single-molecule DNA translocation assay for HELB. (A) Overview of the experimental configuration to measure the translocation of HELB and HELB variants using the C-Trap system. Single 22.2-knt ssDNA molecules were tethered between two streptavidin-coated beads held in dual optical traps. A confocal laser scanned the DNA tethers to visualize HELB molecules labelled with a quantum dot. (B) Schematic of the microfluidic flow cell employed in the optical tweezers measurements. Individual tethers were assembled in channels 1-3 under laminar flow conditions, with separate channels containing streptavidin-coated beads (1), DNA for ssDNA formation (2), and low-ionic-strength buffer (3). Once a ssDNA tether is formed, the traps were transferred to channel 4 for protein loading and fluorescent imaging. (C) Representative experiment showing HELB activity on ssDNA. Kymograph displaying translocating HELB trajectories (upper panel) and the corresponding DNA extension and force traces showing looping events (lower panel). The loop extrusion velocity was determined by linear regression of the segments corresponding to the gradual decrease in

bioRxiv preprint doi: https://doi.org/10.64898/2026.07.16.738708; this version posted July 17, 2026. The copyright holder for this preprint (which was not certified by peer review) is the author/funder, who has granted bioRxiv a license to display the preprint in perpetuity. It is made available under a CC-BY 4.0 International license.

DNA extension (v_i). Loop size was calculated as the difference between the initial extension and the minimum extension reached during a looping event (Ext_0-Ext_F). (D) Frequency of loop formation observed in the presence of HELB and HELB variants. (E) and (F) Loop extrusion velocity and loop size measured for 5 nM HELB and HELB variants on bare ssDNA. For the interval scatter plots, the central bar represents the mean and the error bars indicate the SD. Sample sizes are indicated below the plots.

bioRxiv preprint doi: https://doi.org/10.64898/2026.07.16.738708; this version posted July 17, 2026. The copyright holder for this preprint (which was not certified by peer review) is the author/funder, who has granted bioRxiv a license to display the preprint in perpetuity. It is made available under a CC-BY 4.0 International license.

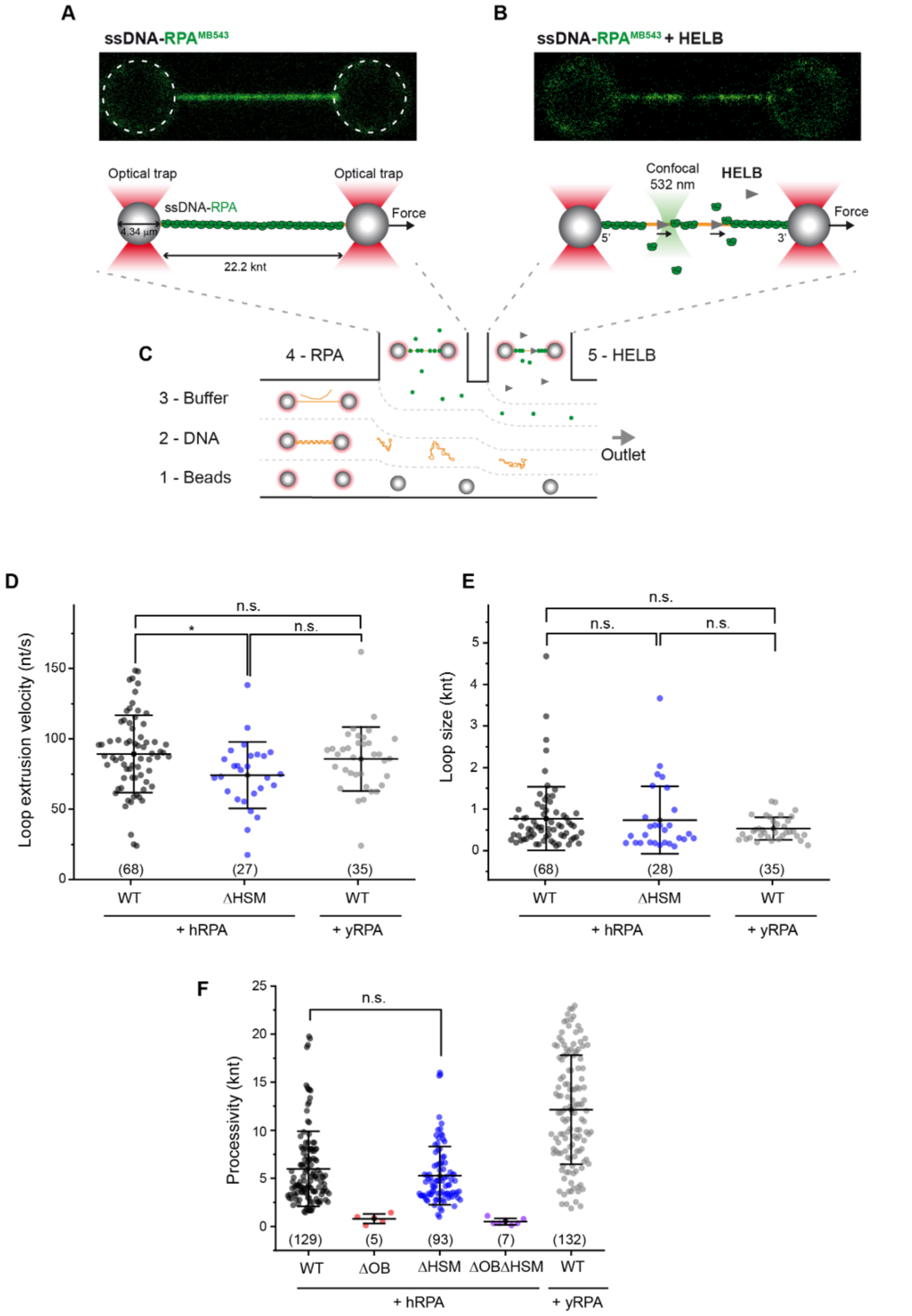

Figure S4: Schematic illustration of the single-molecule RPA clearance assay. (A) Confocal scan (upper panel) and schematic (lower panel) showing fluorescently labelled RPA bound to ssDNA tethered between two optically trapped beads in the C-Trap. The scan was acquired after 2 min in channel 4 containing 15 nM RPA-MB543. (B) The effect

bioRxiv preprint doi: https://doi.org/10.64898/2026.07.16.738708; this version posted July 17, 2026. The copyright holder for this preprint (which was not certified by peer review) is the author/funder, who has granted bioRxiv a license to display the preprint in perpetuity. It is made available under a CC-BY 4.0 International license.

of HELB activity on RPA-ssDNA is monitor in channel 5 containing HELB and ATP but no RPA. (C) Schematic representation of the five-channel fluid cell used in C-Trap experiments. The optical traps are moved sequentially through channels containing a continuously maintained laminar flow according to the following workflow: (1) a pair of micrometre-sized beads is optically trapped in channel 1; (2) a dsDNA tether is immobilized in channel 2; (3) a ssDNA tether is generated by force-induced melting in channel 3; (4) the ssDNA is incubated with RPA in channel 4 containing 15 nM RPA-MB543 for 2 min; and (5) the activity of wild type HELB or HELB variants is measured on ssDNA-RPA tethers in channel 5. (D) and (E) Loop extrusion velocity and loop size for 10 nM HELB and 10 nM ΔHSM on human RPA-ssDNA; and of 10 nM HELB on yeast RPA-ssDNA. (F) Distribution of translocation processivity for 10 nM HELB, ΔOB, ΔHSM and ΔOBΔHSM on human RPA-ssDNA; and for 10 nM HELB on yeast RPA-ssDNA. For the interval scatter plots the center bar represents the mean of the data and the error bars represent the SD. Sample size is indicated below the data.

bioRxiv preprint doi: https://doi.org/10.64898/2026.07.16.738708; this version posted July 17, 2026. The copyright holder for this preprint (which was not certified by peer review) is the author/funder, who has granted bioRxiv a license to display the preprint in perpetuity. It is made available under a CC-BY 4.0 International license.

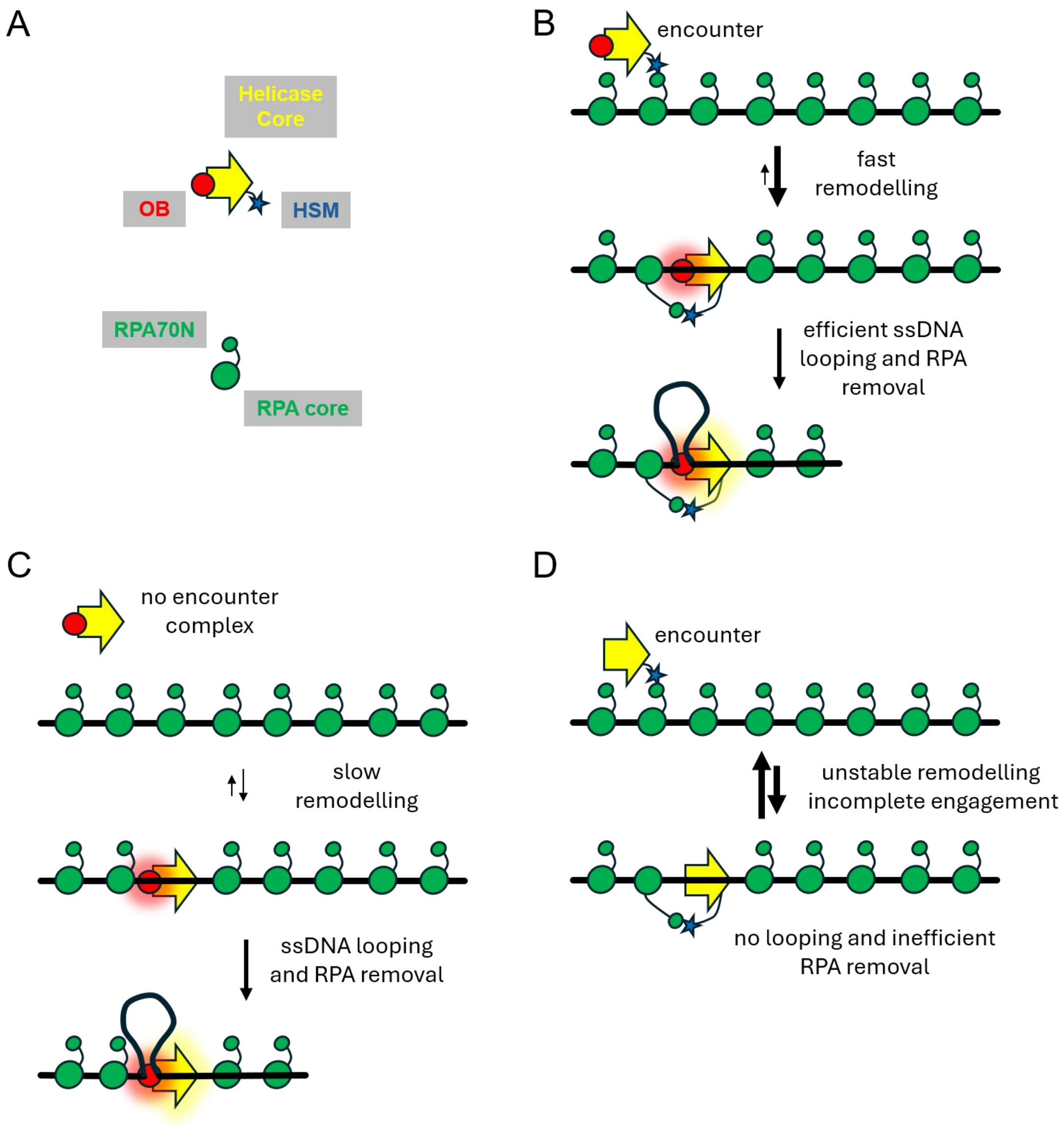

Figure S5: A working model for the effects of OB and HSM domain deletion on HELB recruitment and activation at RPA-ssDNA filaments. (A) Colour coding for domain organisation in cartoon models. (B) Schematic showing a working model for wild type HELB-dependent RPA removal. See also main text and main paper Figure 6 for further detail. (C) The ΔHSM variant is unable to form the initial encounter complex with the exterior of the RPA filament. Therefore, engagement of the core motor and OB anchor domains is slow and inefficient, limiting the overall process, but subsequent translocation, ssDNA looping and RPA removal (when it occurs) is essentially normal. (D) The ΔOB variant can form the initial encounter complex with the exterior of the RPA filament, but remodelling to engage the core motor domains is unstable due to the lack of the OB anchor domain. Failure to form ssDNA loops is associated with very poor RPA removal.
